# Characterization of Bioactive Compounds in Tunisian Bitter Orange (*Citrus aurantium* L.) Peel and Juice and Determination of Their Antioxidant Activities

**DOI:** 10.1155/2013/345415

**Published:** 2013-06-13

**Authors:** Iness Jabri karoui, Brahim Marzouk

**Affiliations:** Laboratoire des Substances Bioactives, Centre de Biotechnologie à la Technopole de Borj-Cédria (CBBC), BP 901, 2050 Hammam-lif, Tunisia

## Abstract

*Citrus aurantium* peel and juice aroma compounds were investigated by gas chromatography (GC) and gas chromatography-mass spectrometry (GC-MS), whereas phenolic compounds analysis was performed by reversed-phase high-performance liquid chromatography (RP-HPLC). Limonene was the major volatile compound of bitter orange peel (90.25%) and juice (91.61%). HPLC analysis of bitter orange peel and juice methanolic extracts indicated that phenolic acids constitute their main phenolic class representing 73.80% and 71.25%, respectively, followed by flavonoids (23.02% and 23.13%, resp.). *p*-Coumaric and ferulic acids were the most abundant phenolic compounds representing 24.68% and 23.79%, respectively, in the peel, while the juice contained 18.02% and 19.04%, respectively. The antioxidant activities of bitter orange peel and juice methanolic extracts have been evaluated using four * in vitro* assays, and the results were compared with the standard antioxidants (BHT, BHA, and ascorbic acid). Our findings demonstrated that *Citrus aurantium* peel and juice possess antioxidant activities which were less effective than those of antioxidant standards. Both extracts may be suggested as a new potential source of natural antioxidant.

## 1. Introduction

Antioxidants have been widely used as food additives to provide protection against oxidative degradation of foods [1]. The most commonly used synthetic antioxidants are butylated hydroxyanisole (BHA), butylated hydroxytoluene (BHT), Propyl gallate (PG), and butylated hydroquinone. However, these synthetic antioxidants have side effects such as liver damage and carcinogenesis [2]. Therefore, there is a need for isolation and characterization of natural antioxidant having less or no side effects, for use in foods or medicinal materials in order to replace synthetic antioxidants [3]. The importance of aromatic plants as natural antioxidants has been well established [4, 5].

The genus *Citrus* of the family Rutaceae includes several important fruits such as oranges, mandarins, limes, lemons, sour orange, and grapefruits. *Citrus* fruits are one of the important horticultural crops, with worldwide agricultural production of over 80 million tons per year [6]. Although the fruits are mainly used for dessert, they have important economic value for their essential oils. *Citrus* essential oils are obtained as byproducts of the *Citrus* processing and are the most widely used essential oils in the world. In fact, *Citrus* fruit essential oils and their major components have gained acceptance in the food industry since they have been generally recognized as safe [7]. *Citrus* essential oils have a wide range of uses. Primarily, they are used as aroma flavour in many food products, including alcoholic and nonalcoholic beverages, marmalades, gelatins, sweets, soft drinks, ice creams, dairy products, oils, candies, and cakes [8–10]. In pharmaceutical industries, they are employed as flavouring agents to mask unpleasant tastes of drugs. Additionally, in perfumery and cosmetics, the low volatile essential oil components play an important role as head notes [8]. 

In Tunisia, citriculture has existed traditionally, and the *Citrus* varieties have been naturally selected. The area under *Citrus* cultivation was estimated to be about 19.250 ha with a yearly production of over 230,000 tons. Such high production inevitably satisfies the fresh fruit market, the agrofood industry, and the exportation demands. Sour orange, known as bitter orange, is a well-known *Citrus* rootstock in Tunisia. Due to its sour and bitter taste, it has not been used as an edible fruit. The juice of the fruit is used in salads for sour taste instead of lemon juice, and the peel is used in jam production [11]. Recently, much attention has been paid to the possible health benefits of dietary phenolics that have antioxidant activities stronger than those of vitamin C. In fact, consumption of *Citrus* fruit or juice is found to be inversely associated with several diseases [12]. Knekt et al. [13] reported that intake of orange resulted in reducing incidence of asthma in Finland. *Citrus *fruit extracts are also found to have several inhibitory activities, such as anti-inflammatory, antitumor, antifungal, and blood clot inhibition activities [14]. The health benefits of *Citrus* fruit have mainly been attributed to the presence of bioactive compounds, such as phenolics (e.g., flavanone glycosides, hydroxycinnamic acids) [15], vitamin C [13], and carotenoids [16]. However, there is hardly any data on the essential oil and phenolics of Tunisian *Citrus *species. Also, to the best of our knowledge, there has been very limited research into the antioxidant capacities of sour orange peel and juice. Therefore, the aim of this research is to study the bioactive contents of Tunisian *Citrus aurantium* juice and peel and to evaluate the antioxidant capacity of their extracts.

## 2. Materials and Methods

### 2.1. Chemicals

All solvents used in the experiments (diethyl ether, chloroform, hexane, acetonitrile, and methanol) were purchased from Merck (Darmstadt, Germany). Sodium methylate (CH_3_ONa), sodium chloride (NaCl), sulphuric acid (H_2_SO_4_), β-carotene, linoleic acid, butylated hydroxyltoluene (BHT), butylated hydroxyanisole (BHA), ethylenediaminetetraacetic acid (EDTA), gallic acid, 2,2-Diphenyl-1-picrylhydrazyl (DPPH), trichloroacetic acid, iron(II)chloride (FeCl_2_), iron(III)chloride anhydrous (FeCl_3_), ascorbic acid, Kaliumhexacyanoferrat(III), and potassium ferricyanide (K_3_Fe(CN)_6_) were obtained from Sigma-Aldrich (Steinheim, Germany). Essential oil standards were purchased from Fluka and Sigma-Aldrich (Steinheim, Germany). Homologous series of C_8_–C_22_ n-alkanes used for identification were obtained from Sigma-Aldrich (Steinheim, Germany). Authentic standards of phenolic compounds were purchased from Sigma and Fluka. Stock solutions of these compounds were prepared in HPLC grade methanol. These solutions were wrapped in aluminium foil and stored at 4°C. All other chemicals used were of analytical grade.

### 2.2. Plant Material

Bitter orange (*Citrus aurantium *L.) is a plant that belongs to the Rutaceae family. Its fruit pulp is acidic and the albedo is bitter [17]. Fresh fruits used in our research come from an orchard located at the Cap-Bon region (North-East of Tunisia); they have been harvested from four selected trees, peeled, and pressed, and the peel and juice were used fresh. 

### 2.3. Essential Oil Extraction

Three lots of 100 g of fresh peel were separately hydrodistilled for 90 min (time kept constant after a kinetic survey during 30, 60, 90, 120, and 150 min). The volatile compounds of the oil were collected in diethyl ether using liquid-liquid isolation. All experiments were done in triplicates, and results were expressed in percentages. 

Juice aroma was extracted according to the protocol described by Tønder et al. [18]. In order to survey the juice aroma content of *Citrus* fruits, 30 g of juice was extracted with 30 mL of a mixture of ether-pentane: (1 : 1, v : v). A known quantity of 2-undecanone was added as an internal standard. The mixture was extracted under magnetic stirring for 30 min. After standing for 15 min, the sample was frozen at −20°C. When the water phase was frozen, the organic phase was recovered in a quickfit pear-shaped flask provided by a Vigreux column then placed in a water bath regulated at 36°C until concentration of the sample to a minimum volume of about 100 mL.

### 2.4. Gas Chromatography-Flame Ionization Detection (GC-FID)

Peel and juice volatiles were analysed by GC using a Hewlett-Packard 6890 apparatus (Agilent technologies, Palo Alto, CA, USA) equipped with a flame ionization detector (FID) and an electronic pressure control (EPC) injector. A polyethylene glycol capillary column (HP INNOWax, 30 m × 0.25 mm i.d, 0.25 *μ*m film thickness) was used; the flow of the carrier gas (N_2_, U) was 1.6 mL min^−1^, and the split ratio was 60 : 1. Analyses were performed using the following temperature program: oven temperature isotherm at 35°C for 10 min, from 35 to 205°C at the rate of 3°C min^−1^, and isotherm at 205°C over 10 min. Injector and detector temperatures were held, respectively, at 250 and 300°C. Surfaces of peaks and percentages of the different compounds were determined using the same HP chemStation cited above.

### 2.5. Gas Chromatography-Mass Spectrometry (GC-MS)

GC-MS analyses were carried out on a gas chromatograph, an HP 5890 series (II) coupled to an HP 5972 mass spectrometer (Agilent Technologies, Palo Alto, CA, USA) with electron impact ionization (70 eV). An HP-5MS capillary column (30 m × 0.25 mm, 0.25 *μ*m film thickness Agilent Technologies, Hewlett-Packard, CA, USA) was used. Column temperature was programmed to rise from 50°C to 240°C at a rate of 5°C/min. The carrier gas was helium with a flow rate of 1.2 mL/min and a split ratio of 60 : 1. Scan time and mass range were 1 s and 40–300 *m/z*, respectively. 

### 2.6. Compounds Identification

Volatile components were identified by comparison of their retention indices (RI) relative to (C_8_–C_22_) n-alkanes with those of the literature or with those of authentic compounds available in our laboratory. Further identification was made by matching their recorded mass spectra with those stored in the Wiley/NBS mass spectral library of the GC/MS data system and other published mass spectra [19]. Determination of the component percentages was based on peak area normalization without using correction factors. Analyses were performed in triplicate.

### 2.7. Polyphenol Extraction

Fresh peels were dipped in liquid nitrogen according to Ghasemi et al. [20] method and ground into a fine powder using a prechilled mortar and pestle. Triplicate subsamples of 1 g of ground peel were extracted by stirring with 10 mL of pure methanol for 30 min. The extract was then kept for 24 h at 4°C, filtered through a Whatman number 4 filter paper, evaporated under vacuum to dryness, and stored at 4°C until analyzed [21]. Extracts obtained will serve for the quantification of polyphenols components and the evaluation of antioxidant activities.

One millilitre of juice was extracted with 9 mL of 80% methanol for 30 min at room temperature according to Xu et al. [22]. After centrifugation at 3000 ×g for 10 min, the supernatant was taken out for determination of polyphenols components and antioxidant capacity. 

### 2.8. Reversed-Phase (RP-HPLC) Analysis and Identification of Phenolic Compounds

For HPLC analysis, 500 *μ*L of resorcinol (1 mg mL^−1^), as internal standard, was added to the methanolic extract. The phenolic compound analysis was carried out using an Agilent Technologies 1100 series liquid chromatograph (RP-HPLC) coupled with a UV-vis multiwavelength detector. The separation was carried out on a 250 × 4.6 mm, 4 *μ*m Hypersil ODS C18 reversed phase column at ambient temperature. The mobile phase consisted of acetonitrile (solvent A) and water with 0.2% sulphuric acid (solvent B). The flow rate was kept at 0.5 mL/min. The gradient programme was as follows: 15% A/85% B 0–12 min, 40% A/60% B 12–14 min, 60% A/40% B 14–18 min, 80% A/20% B 18–20 min, 90% A/10% B 20–24 min, and 100% A 24–28 min [23]. The injection volume was 20 *μ*L, and peaks were monitored at 280 nm. Samples were filtered through a 0.45 *μ*m membrane filter before injection. Phenolic compounds were identified according to their retention times as well as by spiking the sample with standards. Analyses were performed in triplicate.

### 2.9. Total Antioxidant Capacity

The assay is based on the reduction of Mo(VI) to Mo(V) by a flower methanolic extract and subsequent formation of a green phosphate/Mo(V) complex at acid pH [24]. An aliquot of methanolic extract was combined in an Eppendorf tube with 1 mL of reagent solution (0.6 M sulfuric acid, 28 mM sodium phosphate, and 4 mM ammonium molybdate). The tubes were incubated in a thermal block at 95°C for 90 min. After the mixture had cooled to room temperature, the absorbance of each solution was measured at 695 nm (Anthelie Advanced 2, SECOMAN) against a blank solution. The antioxidant capacity was expressed as mg gallic acid equivalents per gram of fresh weight for the peel extract (mg GAE/g of FW) and per liter for the juice extract (mg GAE/L). All samples were analyzed in three replications.

### 2.10. DPPH Assay

The electron donation ability of the obtained extract was measured by bleaching of the purple-coloured solution of 1,1-diphenyl-2-picrylhydrazyl radical (DPPH) according to the method of Hatano et al. [25]. A total of 1 mL of different methanolic extracts concentrations (1, 10, 20, and 100 *μ*g/mL) were added to 0.5 mL of a 0.2 mmol/L DPPH methanolic solution. The mixture was shaken vigorously and left standing at room temperature for 30 min. The absorbance of the resulting solution was then measured at 517 nm after 30 min. The antiradical activity (three replicates per treatment) was expressed as IC_50_ (concentration required to cause a 50% DPPH inhibition: *μ*g/mL) for the peel extract and as *I*% for the juice extract. The ability to scavenge the DPPH radical was calculated using the following equation:

(1) DPPH  scavenging  effect  (%)=[(A0−A1)A0]×100,

where *A*_0_ is the absorbance of the control at 30 min and *A*_1_ is the absorbance of the sample at 30 min. BHT was used as a positive control. All samples were analyzed in triplicate.

### 2.11. Reducing Power Ability

In this assay, the yellow colour of the test solution changes to green depending on the reducing power of test specimen. The presence of reductants in the solution causes the reduction of the Fe^3+^/ferricyanide complex to the ferrous form. Therefore, Fe^2+^ can be monitored by the measurement of the absorbance at 700 nm [26]. The method of Oyaizu [27] was used to assess the reducing power of peel and juice extracts. A total of 1 mL of different extracts concentrations (20, 100, 200, and 500 *μ*g/mL) were mixed with 2.5 mL of a 0.2 M sodium phosphate buffer (pH 6.6, prepared from 62.5 mL of a 0.2 M Na_2_HPO_4_, 37.5 mL of 0.2 M NaH_2_PO_4_·H_2_O), and 2.5 mL of 1% K_3_Fe(CN)_6_, and incubated in a water bath at 50°C for 20 min. Then, 2.5 mL of 10% trichloroacetic acid was added to the mixture that was centrifuged at 650 g for 10 min. The supernatant (2.5 mL) was then mixed with 2.5 mL distilled water and 0.5 mL of 0.1% ferric chloride solution. The intensity of the blue-green colour was measured at 700 nm. The EC_50_ value (mg/mL) is the extract concentration at which the absorbance was 0.5 for the reducing power and was calculated from the graph of absorbance at 700 nm against extract concentration. Ascorbic acid was used as positive control. Tests were carried out in triplicate.

### 2.12. β-Carotene Bleaching Test

A slightly modified Koleva et al. [28] method was employed to estimate sample methanolic extract capacity to inhibit the β-carotene bleaching. β-carotene (2 mg) was dissolved in 20 mL chloroform, and to 4 mL of this solution, linoleic acid (40 mg) and Tween 40 (400 mg) were added. Under vacuum at 40°C, the chloroform was evaporated, and 100 mL of oxygenated water was added, and then the emulsion was vigorously shaken. Reference compounds (BHT and BHA) and samples extracts were prepared in methanol. An aliquot (150 *μ*L) of the β-carotene/linoleic acid emulsion was distributed in each of the wells of 96-well microtiter plates, and methanolic solutions of the test samples (10 *μ*L) were added. Three replicates were prepared for each of the sample concentration. The microtiter plates were incubated at 50°C for 120 min, and the absorbance was measured at 470 nm using a microtiter reader (model EAR 400, Labsystems Multiskan MS). Readings of all samples were performed immediately (*t* = 0 min) and after 30 min or 120 min of incubation. BHT and BHA were used as standard antioxidants. The antioxidant activity (%) of the peel and juice extracts was evaluated in terms of β-carotene bleaching inhibition using the following formula:

(2) $$\begin{array}{c} \%\;\text{Inhibition}=\left[\frac{\left(A_{t}-C_{t}\right)}{\left(C_{0}-C_{t}\right)}\right]\times 100, \end{array}$$

where *A*_*t*_ and *C*_*t*_ are the absorbance values measured for the test sample and control, respectively, after incubation for 120 min, and *C*_0_ is the absorbance values for the control measured at zero time during the incubation. The results are expressed as IC_50_ values (concentration required to cause a 50% *β*-carotene bleaching inhibition: mg/mL) for the peel extract and as %*I* for the juice extract. Tests were carried out in triplicate.

### 2.13. Statistical Analysis

All data were reported as means ± standard deviation of three samples. Statistical analysis was performed with STATISTICA [29]. Differences were tested for significance by the ANOVA procedure, using a significance level of *P* ≤ 0.05.

## 3. Results and Discussions

### 3.1. Volatile Compounds of Bitter Orange Peel and Juice

Volatile compounds of *Citrus aurantium *peel and juice, their retention indexes and percentages, were listed in Table 1. All the constituents were arranged in order of their elution on the HP-5 column, although the retention indexes of compounds confirmed on HP INNOWax column have been also included.

Twenty-seven components were identified in the *Citrus aurantium *peel essential oil, amounting to 99.48% of the total oil. Peel essential oil was dominated by monoterpene hydrocarbons (93.49%), and limonene was the major constituent (90.25%) followed by *α*-terpinene (1.10%). Linalool was the main oxygenated monoterpenes (1.56%) of the essential oil. 

Twelve compounds were identified in the juice representing 99.56% of the total aroma. As with sour orange peel, juice aroma comprised mainly monoterpene hydrocarbons with limonene (91.61%), *α*-phellandrene (1.84%), and *α*-thujene (1.03%) being the major components. Among oxygenated sesquiterpenes, the main component was caryophyllene oxide (1.42%). Our data were in concordance with the limonene chemotype of *Citrus aurantium* growing in Tunisia (stage 3: mature), previously reported by Saidani and Marzouk [30], Tounsi et al. [31], and Hosni et al. [32].

Previous compositional studies regarding the peel oil and juice aroma constituents of sour orange showed similar results proving that limonene was the major volatile compound. Indeed, Boussaada and Chemli [33] have reported that the content of limonene in Tunisian sour orange var. Amara varied from 87% to 92.2% on fresh weight basis. These values are lower than those obtained herein. Report from Italy has shown that limonene (94.3%) followed by myrcene (1.88%), linalool (0.78%), and *α*-pinene (0.4%) was the chief components of sour orange [34]. More recently, Moraes et al. [35] have investigated the peel oil composition of Brazilian *Citrus aurantium *without specifying the variety and found that limonene (97.5–98%), myrcene (1.2–1.45%), and octanol (0.34–0.54%) were the prominent compounds.

Several investigations have confirmed the limonene importance in *Citrus* juice [36, 37]. Moufida and Marzouk [30] showed that limonene was the major volatile component of four *Citrus* species representing 63% in blood orange and 90% in sour orange. In addition, Tønder et al. [18], reported that limonene was the most abundant compound in orange juice (88.9%) which is in agreement with our results. Tounsi et al. [31] showed that limonene was highly represented in mandarin juice (69.59%), blood orange (57.65%), and bitter orange (48.85%). This latter value is lower than that obtained herein. 

Limonene is one of the most common terpenes in nature and is the major constituent of an essential oil series. Its pleasant citric fragrance is commonly used as a flavoring in foods and drinks [38], for which it is classified in the U.S. Code of Federal Regulation as safe. According to Moraes et al. [35], besides being popular flavoring agents found in common food items, the essential oil from *C. aurantium *and its major constituent limonene present substantial antiulcerogenic and gastroprotective actions that can be regarded as a promising target for the development of a new drug for the prevention of gastric ulcer. Tests on animals have proven the effectiveness of limonene against some types of cancer including gastric, mammary, pulmonary adenoma, and liver [38]. Besides the effectiveness of limonene in traditional medicine to treat severe dermatitis, fatigue recovery [39], and depression [40], different studies have reported its anxiolytic and antidepressant effects on the central nervous system [41].

### 3.2. Identification and Quantification of Phenolic Compounds by RP-HPLC

**Figure 1:**
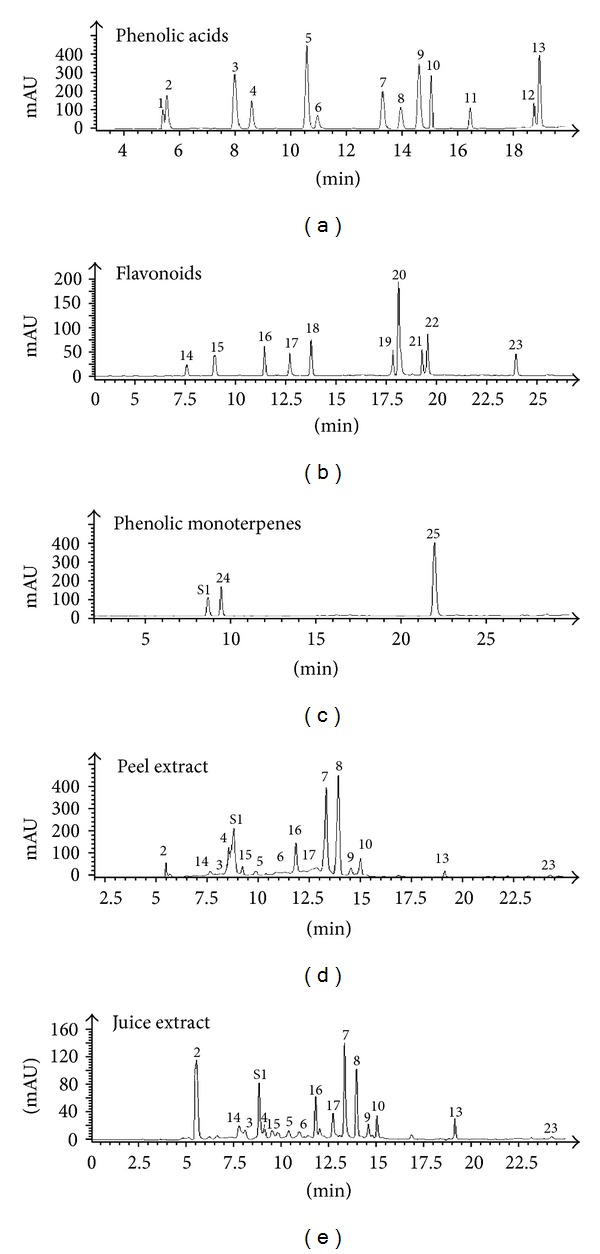
RP-HPLC chromatographic profiles of phenolic acids, flavonoids, and phenolic monoterpenes standards and *Citrus aurantium *peel and juice extracts monitored at 280 nm. *The peak numbers correspond to the following: 1. caffeic acid; 2. gallic acid; 3. *p*-hydroxybenzoic acid; 4. chlorogenic acid; 5. syringic acid; 6. vanillic acid; 7. *p*-coumaric acid; 8. ferulic acid; 9. trans-2-hydroxycinnamic acid; 10. rosmarinic acid; 11. *o*-coumaric acid; 12. cinnamic acid; 13. trans-cinnamic acid; 14. epicatechin gallate; 15. catechin; 16. rutin; 17. naringin; 18. quercetrin; 19. luteolin; 20. quercetin; 21. apigenin; 22. amentoflavone; 23. flavone; 24. tyrosol; 25. thymol; S1. resorcinol.

Polyphenol qualitative and quantitative determination of *Citrus aurantium *peel and juice was performed by RP-HPLC analysis. The chromatogram of bitter orange peel and juice methanolic extracts as compared to authentic standards of phenolic acid, flavonoid, and phenolic monoterpenes profiles (Figure 1) allowed to identify fifteen phenolic compounds including ten phenolic acids (gallic, vanillic, *p*-coumaric, chlorogenic, syringic, rosmarinic, trans-2-hydroxycinnamic, ferulic, *p*-coumaric, and trans-cinnamic acids), five flavonoids (gallate epicatechin, catechin, rutin, naringin, and flavone) in the peel extract, nine phenolic acids (gallic, hydroxybenzoic, syringic, vanillic, rosmarinic, trans-2-hydroxycinnamic, trans-cinnamic, and *p*-coumaric and ferulic), five flavonoids (epicatechin, catechin, rutin, naringin, and flavone), and one phenolic alcohol (tyrosol) in the juice extract (Figure 1). The main phenolic class of the peel and juice extracts (Table 2) was phenolic acids representing 73.8% (1.03 mg/g) and 71.25% (473.89 mg/L), respectively, followed by flavonoids (23.02%; 0.33 mg/g of the peel) and (23.13%; 136.91 mg/L of juice). *p*-Coumaric and ferulic acids were the most abundant phenolic compounds of *Citrus aurantium* representing 24.68% and 23.79%, respectively, in the peel extract and 18.02% and 19.04%, respectively, in the juice. Among flavonoids, rutin was the most abundant compound constituting 9.91% of the peel extract phenols and 5.98% of the juice. 

Bocco et al. [42] reported similar results showing the prevalence of phenolic acids in *Citrus* fruits. In addition, Robbins [43] found that phenolic acids (caffeic, *p*-coumaric, ferulic, and sinapic) are characteristic of *Citrus.* Similarly, Gorinstein et al. [44] found that ferulic, sinapic, *p*-coumaric, and caffeic acids amounts were significantly higher in the peel than in the fruit peeled. Generally, the methanol extracts of *Citrus* peels are known for their richness in phenolic compounds, such as phenolic acids and flavonoids [45]. Moreover, Anagnostopoulou et al. [46] reported that the peel was the richest part in flavonoids of *Citrus *fruits. Previous studies have also shown the presence of higher contents of phenolic compounds in fruit peel than segment of *Citrus* [44, 47].

Our results are also comparable to those of Tounsi et al. [31] who confirmed the richness of *Citrus* juice in phenolic acids, which constitute 86.4% of the bitter orange juice TP, 73.13% of the mandarin, 83.90% of blood orange juice, and 74.52% of the lemon. These authors found that ferulic (20.42%), vanillic (19.76%), rosmarinic (18.7%), and *p*-coumaric (15.10%) acids were the major phenolic compounds of bitter orange juice. In addition, Robbins [43] found that phenolic acids (caffeic, *p*-coumaric, ferulic, and sinapic) are characteristic of *Citrus.* Furthermore, Caro et al. [48] and Gorinstein et al. [49] mentioned that in addition to vitamin C and carotenoids, a variety of phenolic compounds, namely, flavanone glycosides, and hydroxycinnamic acids, are present in *Citrus* fruit as bioactive compounds. Naringin and hesperidin, so-called *Citrus* flavonoids, are two major flavanone glycosides present in *Citrus* fruits [48, 50]. All flavonoids described in *Citrus* sp. can be classified into these groups: flavanones, flavones, and flavonols, and species are characterized by a particular flavanone glycoside pattern. In fact, *Citrus* plants contain a wide range of flavonoid constituents, some of which are characteristic of them [51]. It is important to note that the *Citrus* flavonoids have been found to have health-related properties, which include anticancer, antiviral, and anti-inflammatory activities, effects on capillary fragility, and an ability to inhibit human platelet aggregation [52, 53].

Several studies showed that phenolics exhibit a wide range of biological effects including antibacterial, anti-inflammatory, antiallergic, hepatoprotective, antithrombotic, antiviral, anticarcinogenic, and vasodilatory actions [54, 55]. For instance, phenol carboxylic acids such as caffeic, chlorogenic, *p*-coumaric, and ferulic acids exert beneficial effects on human health through prevention of degenerative pathologies such as cardiovascular diseases and cancer [56]. Zang et al. [57] showed that *p*-coumaric acid can act as a direct scavenger of reactive oxygen species to prevent lipid peroxidation, reduce serum cholesterol levels, and enhance the resistance of LDL to oxidation. In addition, Srinivasan et al. [58] reported that ferulic acid exhibits a wide range of therapeutic effects against various diseases like cancer, diabetes, cardiovascular, and neurodegenerative diseases. A wide spectrum of beneficial activity for human health has been advocated for this phenolic compound, at least in part, because of its strong antioxidant activity.

### 3.3. Antioxidant Activities

Considering the multifaceted aspects of antioxidants and their reactivity, several antioxidant assays were applied. In fact, depending on the reaction involved, these assays can roughly be classified into two types: assays based on hydrogen atom transfer reactions and assays based on electron transfer [59]. The result of a single method can give only a reductive view of the antioxidant properties of the extracts [60]. In fact, the antioxidant activity may be due to different mechanisms, such as prevention of chain initiation, decomposition of peroxides, and prevention of continued hydrogen abstraction, free radical scavenging, reducing capacity, and binding of transition metal ion catalysts [61]. Thus, to evaluate antioxidant effectiveness, several analytical methods and different substrates are used. 

Total antioxidant capacity by phosphomolybdenum method assay is based on the reduction of Mo(VI) to Mo(V) by the sample analyte and the subsequent formation of green phosphate/Mo(V) complex at acidic pH [3]. The phosphomolybdenum method is quantitative since the total antioxidant activity is expressed as the number of equivalents of gallic acid. The measure of the global antioxidant capacity considers the cumulative action of all the antioxidants present in plant extract, thus providing an integrated parameter rather than the simple sum of measurable antioxidants. The total antioxidant capacity of plant extracts takes into account all antioxidants and synergistic effects between them. In addition, the antioxidant capacity of phenolic compounds is mainly due to their redox properties, which can play an important role in absorbing and neutralizing free radicals, quenching singlet and triplet oxygen, or decomposing peroxides [62]. 

Total antioxidant capacities of bitter orange peel and juice methanolic extracts were presented in Table 3. Our study revealed a total antioxidant capacity of 5.23 mg GAE/g in the peel extract and 823.13 mg GAE/L in the juice. To the best of our knowledge, there are no data concerning bitter orange peel and juice total antioxidant capacities using phosphomolybdenum method. The results of the statistical analysis showed a highly significant positive correlation between total antioxidant activity and flavonoid content of the peel and juice methanolic extracts (*r* = 0.99, *r* = −0.98 at *P* < 0.05, resp.). 

A similar correlation (*r* = 0.82, *P* < 0.05) was observed by Abeysinghe et al. [47] in four species of *Citrus* which confirms the close relationship between the antioxidant activity and total flavonoid contents. Our results are also in agreement with those reported by Patil et al. [63] who showed that the antioxidant activity of *Citrus aurantifolia* juice is due to the presence of flavonoids, phenolic compounds, and ascorbic acid. Peel and juice antioxidant activity might be attributed to the presence of phytochemicals such as phenolic compounds. In fact, several studies [11, 64] attributed antioxidant activities to the presence of phenolic compounds in *Citrus.* Thus, *Citrus* fruits have received much attention because of their nutritional and antioxidant properties, and nowadays prevention of health problems through nutrition is promoted intensively [46], due mainly to the contribution of antioxidant compounds including vitamin C, phenolic compounds, and carotenoids. Cano et al. [65] mentioned that in *Citrus,* the major part of the total antioxidant activity is due to the hydrophiliccompound, and some authors have stressed the main role of hesperidin in the total antioxidant capacity of orange juices [66]. Moreover, *Citrus* species of various origins have been assessed for their phenolic constituents and antioxidant activities [46]. Indeed, *Citrus* fruit extracts and *Citrus* flavonoids exhibit a wide range of promising biological properties including antiatherogenic, anti-inflammatory, antitumor activities, and strong antioxidant capacity [67]. Previous studies have also shown that *Citrus* fruits are rich in phenolic compounds and have high antioxidant activity [64, 68].

Furthermore, Singh et al. [69] reported that monoterpenic essential oils are considered as natural antioxidants. Similarly, Zia-ur-Rehman [70] found that *Citrus* fruit byproducts could be interesting not only for their important fibre content but also because of their antioxidant capacity. They have high fibre and vitamin contents as well as other associated bioactive compounds such as flavonoids and terpenes which exhibit interesting antioxidant properties [71]. 

DPPH can be used to determine the free radical scavenging activity as it forms a stable molecule on accepting an electron or hydrogen atom [72]. There was a reduction in the concentration of DPPH due to the scavenging effect of extracts. The extracts and standard antioxidants reduced DPPH to yellow coloured product in a concentration-dependent manner.

Free radical scavenging properties of methanolic extract of bitter orange peel and juice are presented in Table 3. Peel methanolic extract showed higher IC_50_ value (190 *μ*g/mL) than that of the standard antioxidant BHT (IC_50_ = 25 *μ*g/mL) indicating a low antioxidant capacity strongly correlated with the TF content (*r* = 0.99, *P* < 0.05). Tumbas et al. [73] reported a comparable radical scavenging activity (IC_50_ = 179 *μ*g/mL) when studying the peel extract of mandarin (*Citrus reticulata*). However, Ghasemi et al. [20] reported that the *Citrus aurantium* fruits peel collected in Iran was characterized by a lower antiradical activity (IC_50_ = 1.9 mg/mL). In addition, Muthiah et al. [74] showed that Indian *Citrus aurantium* peel was characterized by a higher antiradical activity (IC_50_ = 86.83 *μ*g/mL). Moreover, Ersus and Cam [11] reported that Turkish sour orange peel-free has higher antiradical activity (IC_50_ = 0.993 mg/mg).

On the other hand, juice methanolic extract was characterized by inhibition percentages of 97.05%. Our results are comparable to those of Tounsi et al. [31] who showed in their study on the antioxidant activity of the juice of four *Citrus* varieties that bitter orange juice has a high antiradical activity compared to blood orange, lemon, and mandarin (96.1%, 90.21%, 63.8%, and 56.75%, resp.). In addition, Patil et al. [63] reported a comparable radical scavenging activity (85.4 and 90%) in the chloroform extract of *Citrus aurantifolia* juice. The statistical analysis shows a highly significant correlation between antiradical activity and TP content of juice extract (*r* = −0.99, *P* < 0.05). These results are similar to those of Amić et al. [75] who reported a correlation between the phenolic content and antioxidant capacity of plant foods. According to Rangkadilok et al. [76] and Patil et al. [63], the antiradical activity of peel, juice, and pulp is partly attributed to phenolic compounds such as gallic acid and rutin which have high antiradical activity.

In addition, according to Kumaran and Joel Karunakaran [77], antioxidant molecules such as polyphenols, flavonoids, and tannins reduce and discolour DPPH due to their hydrogen donating ability. Moreover, Tounsi et al. [31] mentioned that bitter orange and blood orange juices displayed higher activity than lemon and mandarin (values were 96.1%, 90.21%, 63.8%, and 56.75%, resp.).

The free radical scavenging activity of fruit extracts [78] has been extensively studied. Essential oils rich in monoterpenes are recognized as food preservatives, and monoterpenic essential oils are natural antioxidants that are active against certain cancers [79]. Indeed, a number of dietary monoterpenes have antitumoral activity that can prevent the formation or progress of cancer and cause tumor regression. Limonene has a well-established protective activity against many types of cancer [80]. In addition, the phenolic acids exhibited radical scavenging activities in the order of gallic > gentisic > syringic > caffeic > protocatechuic > sinapic > ferulic > isoferulic > vanillic > *p-*coumaric > *o-*coumaric > *m-*coumaric > salicylic ≫ *p-*hydroxybenzoic. Gallic acid, with three hydroxyl groups, was observed to be the most active phenolic acid [81].

Ogiwara et al. [82] previously found that caffeic, chlorogenic, and ferulic acids scavenged various radicals such as superoxide anions and hydroxy radicals.

On the other hand, Kanski et al. [83] reported that ferulic acid is a phenolic compound that possesses three distinctive structural motifs that can possibly contribute to the free radical scavenging capability of this compound. The presence of electron donating groups on the benzene ring (3 methoxy and more importantly 4-hydroxyl) of ferulic acid gives the additional property of terminating free radical chain reactions. The next functionality—the carboxylic acid group in ferulic acid with an adjacent unsaturated C–C double bond—can provide additional attack sites for free radicals and thus prevent them from attacking the membrane. In addition, this carboxylic acid group also acts as an anchor of ferulic acid, by which it binds to the lipid bilayer, providing some protection against lipid peroxidation [83]. 

In the β-carotene linoleate system, β-carotene undergoes rapid discolouration in the absence of antioxidants. This test measures the sample's potential for inhibiting conjugated diene hydroperoxides formation from linoleic acid oxidation [84]. 

Our results (Table 3) showed that the antioxidant activity of the peel extract was very low (IC_50_ = 5.81 mg/mL) compared to antioxidant standards BHT (IC_50_ = 0070 mg/mL) and BHA (IC_50_ = 0.043 mg/mL). This activity is strongly correlated with the flavonoid content (*r* = −0.98, *P* < 0.05). The inhibitory effect of β-carotene by the peel methanolic extracts could be due to polar phenolic compounds such as antioxidants. Indeed, Narayan et al. [85] found that flavonoids can inhibit lipid peroxidation process by scavenging free radicals. To the best of our knowledge, there are no data concerning the β-carotene bleaching inhibition capacity of bitter orange peel extract.

The results in Table 3 proved that the juice extract was characterized by a low antioxidant activity of 15.92%. After statistical analysis, our results showed a highly significant correlation between this antioxidant capacity and TP content (*r* = −0.98, *P* < 0.05). Similar results were found by Tounsi et al. [31] indicating a low antioxidant activity (18.27%) of bitter orange juice compared to lemon (22.67%) and mandarin (26.67%) juices.

The transformation of Fe^3+^ into Fe^2+^ in the presence of various fractions was measured to determine the reducing power ability. The reducing ability of a compound generally depends on the presence of reductones (antioxidants), which exert the antioxidant activity by breaking the free radical chain by donating a hydrogen atom [86]. The reduction of the Fe^3+^/ferricyanide complex to the ferrous form occurs due to the presence of reductants in the solution [87]. Reductones are believed not only to react directly with peroxides but also prevent peroxide formation by reacting with certain precursors. According to Amarowicz et al. [88], reducing power is associated with the antioxidant activity.

Table 3 showed that bitter orange peel methanolic extract exhibited a very little Fe^3+^ reducing power ability since its EC_50_ value (1.88 mg/mL) was far higher than that of ascorbic acid (0.04 mg/mL). However, juice's extract showed no reducing power activity. A highly significant correlation was found between the reducing activity and flavonoid or TP contents (*r* = 0.91 and *r* = −0.74, resp., at *P* < 0.05). Different results were reported by Su et al. [68] who showed greater activity in sour orange peel grown in China (EC_50_ = 0.12 mg/mL). In addition, Muthiah et al. [74] found that Indian *Citrus aurantium* peel and fruit extracts were characterized by a higher reducing power ability of 0.536 and 0.314 *μ*g/mL at the highest concentration tested (800 *μ*g/mL). However, our results were in agreement with those of Ramful et al. [89] who found that TP of the flavedo extract of *Citrus* fruits grown in Mauritius correlated strongly with ferric reducing antioxidant capacity (*r* = 0.88). The antioxidant activity of peel extract might be due to the reduction of superoxide anion, inactivation of free radicals, or complexion with metal ions or their combination. 

In conclusion, our results showed that *Citrus aurantium* essential oil was rich in limonene and that ferulic and *p*-coumaric acids were the main phenolic compounds of the peel and juice methanolic extracts. Moreover, our study can be considered as the first report on the antioxidant capacities based on total antioxidant capacity assay, quantification of polyphenols, and HPLC analysis of phenolics. *Citrus aurantium* peel and juice antioxidant activity was high enough for the plant to be used as a potential resource of natural antioxidants for the food industry so that it is interesting to examine its application as a natural antioxidant additive in some final food products. To understand the antioxidant mechanism of action as bioactive components, further fractionation of methanolic extracts, isolation of phenolic compounds, and determination of their biological activities *in vitro* and *in vivo* are needed.

## Figures and Tables

**Table 1:** Essential oil composition of *Citrus aurantium *peel and juice^A^.

Volatile compounds	RI^a^	RI^b^	Peel	Juice	Identification
*α*-thujene	928	1035	—	1.03 ± 0.04^a^	IR. SM. Co-CG
Tricyclen	930	1015	—	0.68 ± 0.18^a^	IR. Co-CG. SM
*α*-Pinene	939	1032	0.55 ± 0.06^a^	0.55 ± 0.01^a^	IR. Co-CG. SM
β-Pinene	980	1118	0.44 ± 0.04^a^	—	IR. SM. Co-CG
Sabinene	975	1132	0.48 ± 0.16^a^	—	CG-SM
Myrcene	991	1174	0.04 ± 0.00^b^	0.68 ± 0.16^a^	IR. SM. Co-CG
*α*-Phellandrene	1006	1176	—	1.84 ± 0.04^a^	CG-SM
*α*-Terpinene	1018	1188	1.10 ± 0.27^a^	—	IR. CG-SM
Limonene	1030	1203	**90.25** **±** **0.81** ^a^	**91.61** **±** **0.97** ^a^	IR. SM. Co-CG
1-8 Cineole	1033	1213	0.87 ± 0.06^a^	0.31 ± 0.04^b^	IR. SM. Co-CG
E-β-Ocimene	1050	1266	0.36 ± 0.00^a^	—	IR. CG-SM
Terpinolene	1088	1290	0.28 ± 0.01^a^	—	IR. SM. Co-CG
Cis-linalool oxide	1074	1478	0.23 ± 0.03^a^	0.49 ± 0.23^a^	CG-SM
Trans-linalool oxide	1088	1450	0.13 ± 0.01^b^	0.21 ± 0.01^a^	CG-SM
Linalool	1098	1553	1.56 ± 0.08^a^	—	IR. SM. Co-CG
Linalyl acetate	1257	1556	0.29 ± 0.02^a^	—	IR. SM. Co-CG
Bornyl acetate	1270	1590	0.14 ± 0.05^a^	—	CG-SM
Terpinene-4-ol	1419	1612	0.06 ± 0.01^a^	—	CG-SM
β-Caryophyllene	1178	1611	0.05 ± 0.01^a^	—	CG-SM
*γ*-Elemene	1492	1623	0.05 ± 0.00^a^	—	CG-SM
Neral	1240	1694	0.13 ± 0.00^a^	—	CG-SM
β-Farnesene	1456	1696	0.15 ± 0.15^a^	—	CG-SM
*α*-Terpineol	1189	1709	0.56 ± 0.03^a^	0.38 ± 0.08^b^	IR. SM. Co-CG
Neryl acetate	1385	1733	0.23 ± 0.03^b^	0.36 ± 0.06^a^	IR. SM. Co-CG
Δ-Cadinene	1523	1755	0.28 ± 0.02^a^	—	CG-SM
Geranyl acetate	1383	1765	0.11 ± 0.00^a^	—	IR. SM. Co-CG
Nerol	1228	1797	0.08 ± 0.00^a^	—	CG-SM
Geraniol	1255	1857	0.16 ± 0.01^a^	—	IR. SM. Co-CG
E-Nerolidol	1566	2030	0.35 ± 0.04^a^	—	CG-SM
Oxyde de caryophyllène	1581	2008	—	1.42 ± 0.51^a^	IR. CG-SM
E.Z farnesyl acetate	1818	2198	0.57 ± 0.02^a^	—	CG-SM
NI			0.52 ± 0.03	0.44 ± 0.05	

^A^Values are given as mean ± SD (*n* = 3). Values followed by the same letter did not share significant differences at *P* < 0.05 (Duncan's test). —: not detected.

^b^Components are listed in order of elution in apolar column (HP-5).

^c^RI^a^, RI^b^: retention indices calculated using, respectively, an apolar column (HP-5) and polar column (HP INNOWax). Volatile compound proportions were calculated from the chromatograms obtained on the HP INNOWax column.

**Table 2:** Contents (mg/g) and percentages (%) of phenolic compounds of bitter orange peel and juice^A^.

Phenolic compounds	Peel		Juice	
	%	Mg/g	%	Mg/L
Phenolic acids	**73.80** **± 3.33** ^ a^	**1.03** **± 0.02**	**71.25** ± 0.25^a^	**473.89** ** ± 0.30**
Gallic acid	1.84 ± 0.25^b^	0.03 ± 0.01	**13.05** ± 0.01^a^	84.53 ± 0.07
Hydroxybenzoic acid	1.13 ± 0.65^b^	0.02 ± 0.01	4.05 ± 0.11^a^	27.88 ± 0.05
Chlorogenic acid	8.63 ± 0.13^a^	0.12 ± 0.01	—	—
Syringic acid	1.69 ± 0.46^b^	0.02 ± 0.01	2.24 ± 0.00^a^	13.75 ± 0.1
Vanillic acid	1.75 ± 0.57^b^	0.02 ± 0.01	2.61 ± 0.04^a^	17.19 ± 0.01
Rosmarinic acid	5.58 ± 0.65^a^	0.08 ± 0.02	5.43 ± 0.10^a^	36.84 ± 0.02
Trans-2-hydroxycinnamic acid	3.15 ± 0.28^a^	0.04 ± 0.01	4.56 ± 0.05^a^	30.69 **± **0.20
Trans-cinnamic acid	1.56 ± 0.65^b^	0.02 ± 0.01	2.58 ± 0.01^a^	15.64 ± 0.09
*p*-Coumaric acid	**24.68** **± 2.64** ^ a^	0.34 ± 0.01	**18.02** ± 0.22^b^	116.13 ± 0.18
Ferulic acid	**23.79** **± 3.27** ^ a^	0.33 ± 0.02	**19.04** ± 0.22^b^	131.24 ± 0.21
Flavonoids	**23.02** ** ± 3.83** ^ a^	**0.33** **± 0.07**	**23.13** ± 1.11^a^	**136.91** ± 0.17
Epicatechin	2.77 ± 0.83^b^	0.04 ± 0.02	5.36 ± 0.15^a^	36.47 ± 0.05
Catechin	3.17 ± 0.27^a^	0.04 ± 0.01	3.16 ± 0.08^a^	20.24 ± 0.09
Rutin	9.91 ± 1.18^a^	0.14 ± 0.03	5.98 ± 0.19^b^	37.21 ± 0.10
Naringin	5.23 ± 1.15^a^	0.07 ± 0.02	5.59 ± 0.04^a^	35.05 ± 0.12
Flavone	1.95 ± 1.98^a^	0.03 ± 0.03	1.19 ± 0.01^a^	7.96 ± 0.01
Phenolic monoterpenes	—		**3.51** ± 0.17^a^	**23.54** ± 0.03
Tyrosol	—		3.51 ± 0.17^a^	23.54 ± 0.03
Unknown	3.18 ± 0.50	0.04 ± 0.01	3.62	22.79 ± 0.08

Total	100	1.40 ± 0.09	100	657.13 ± 0.27

^A^Values are given as mean ± SD (*n* = 3). Values followed by the same letter did not share significant differences at *P* < 0.05 (Duncan's test). —: not detected.

**Table 3:** Antioxidant activities of bitter orange peel and juice methanolic extracts^A^.

	Total antioxidant capacity (mg GAE/g FW: peel) (mg GAE/L: juice)	DPPH (IC_50_, *µ*g mL^−1^: peel) (%*I*: juice)	β-Carotene bleaching (IC_50_, mg mL^−1^: peel) (%*I*: juice)	Reducing power (EC_50_, mg mL^−1^: peel)
Peel	5.23 ± 0.05	190 ± 0.01	5.81 ± 0.03	1.88 ± 0.05
Juice	823.13 ± 17.18	97.05 ± 0.38	15.92 ± 0.66	—

^A^Values are given as mean ± SD (*n* = 3). —: not detected.
